# Characterization and discrimination of microbial community and co‐occurrence patterns in fresh and strong flavor style flue‐cured tobacco leaves

**DOI:** 10.1002/mbo3.965

**Published:** 2019-12-05

**Authors:** Qianying Zhang, Zongze Geng, Dongliang Li, Zhongyang Ding

**Affiliations:** ^1^ Technical Research Center China Tobacco Sichuan Industrial Co., Ltd. Chengdu China; ^2^ National Engineering Laboratory for Cereal Fermentation Technology School of Biotechnology Jiangnan University Wuxi China

**Keywords:** co‐occurrence patterns, flue‐cured tobacco leaf, Illumina MiSeq sequencing, microbial community, network analysis, partial least squares discriminant analysis

## Abstract

Fermentation, also known as aging, is vital for enhancing the quality of flue‐cured tobacco leaves (FTLs). Aged FTLs demonstrate high‐quality sensory characteristics, while unaged FTLs do not. Microbes play important roles in the FTL fermentation process. However, the eukaryotic microbial community diversity is poorly understood, as are microbial associations within FTLs. We aimed to characterize and compare the microbiota associated with two important categories, fresh and strong flavor style FTLs, and to reveal correlations between the microbial taxa within them. Based on 16S and 18S rRNA Illumina MiSeq sequencing, the community richness and diversity of prokaryotes were almost as high as that of eukaryotes. The dominant microbes of FTLs belonged to seven genera, including *Pseudomonas, Bacillus*, *Methylobacterium*, *Acinetobacter*, *Sphingomonas*, *Neophaeosphaeria*, and *Cladosporium*, of the *Proteobacteria*, *Firmicutes*, and *Ascomycota* phyla. According to partial least square discriminant analysis (PLS‐DA), *Xanthomonas*, *Franconibacter*, *Massilia*, *Quadrisphaera*, *Staphylococcus*, *Cladosporium*, *Lodderomyces*, *Symmetrospora*, *Golovinomyces*, and *Dioszegia* were significantly positively correlated with fresh flavor style FTLs, while *Xenophilus*, *Fusarium*, unclassified *Ustilaginaceae*, *Tilletiopsis*, *Cryphonectria*, *Colletotrichum*, and *Cyanodermella* were significantly positively correlated with strong flavor style FTLs. Network analysis identified seven hubs, *Aureimonas*, *Kocuria*, *Massilia*, *Brachybacterium, Clostridium*, *Dietzia*, and *Vishniacozyma*, that may play important roles in FTL ecosystem stability, which may be destroyed by *Myrmecridium*. FTL microbiota was found to be correlated with flavor style. Species present in lower numbers than the dominant microbes might be used as microbial markers to discriminate different flavor style samples and to stabilize FTL microbial communities. This research advances our understanding of FTL microbiota and describes a means of discriminating between fresh and strong flavor FTLs based on their respective stable microbiota.

## Graphical abstract

In this study, we use 16S and 18S rRNA Illumina MiSeq sequencing data to characterize and compare the microbiota associated with fresh and strong flavor style flue‐cured tobacco leaves and reveal correlations between the microbial taxa within them. Flue‐cured tobacco leaf microbiota are correlated with flavor style, and species present at low numbers within the microbial communities can be used as microbial markers to discriminate between samples of different flavor styles and to stabilize flue‐cured tobacco leaf microbial communities.

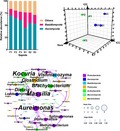

## 1 INTRODUCTION

Tobacco (*Nicotiana tabacum* L.) is one of the largest economic nonfood crops in the world. In China, the most important type of tobacco is the flue‐cured tobacco (Su et al., 2011; Zhao et al., 2007). The flavor of flue‐cured tobacco leaf (FTL) changes throughout the process of fermentation, gradually aging over long periods (typically at least 12 months). The aging process results in FTL of a high commercial quality and causes a change in color to a darker yellow, elimination of harmful odors, degradation of harmful substances, reduction of incentive odor, and development of tobacco‐specific flavors (Yu & Gong, 2009). According to flavor styles, Chinese aged FTL could be traditionally divided into three categories: fresh flavor style, middle flavor style, and strong flavor style.

Microbes have been found to play important roles during the FTL fermentation process (Reid, McKinstry, & Haley, 1937), which include the production of tobacco‐specific flavors (English, Bell, & Berger, 1967) and degradation of nicotine and tobacco‐specific nitrosamines (Gong et al., 2009; Liu, He, et al., 2015; Liu, Ma, et al., 2015). Studies based on traditional culture‐dependent methods have isolated *Bacillus*, *Streptomyces*, *Aspergillus*, and *Penicillium* from FTLs, identifying these species as dominant microbes in FTL fermentations (Qiu, Zhao, Yue, Qi, & Zhang, 2000; Zhao, Qiu, Zhang, Qi, & Yue, 2000). To better characterize the microbial community associated with FTLs, prokaryotic diversity has been investigated using culture‐independent molecular biology techniques such as polymerase chain reaction denaturing gradient gel electrophoresis (PCR‐DGGE) (Huang et al., 2010; Zhao et al., 2007), 16S rRNA gene libraries (Su et al., 2011), Roche 454 bar‐coded pyrosequencing (Liu, He, et al., 2015; Liu, Ma, et al., 2015), and Illumina MiSeq sequencing (Wang et al., 2018). However, little is known about the eukaryotic community structure of FTL based on culture‐independent molecular biology techniques.

Partial least squares discriminant analysis (PLS‐DA) can be used to construct discrimination and classification models by reduction of data dimensionality (Berrueta, Alonso‐Salces, & Héberger, 2007; Vaclavik, Lacina, Hajslova, & Zweigenbaum, 2011), which is a powerful means of discriminating samples with different characteristics (Ramadan, Jacobs, Grigorov, & Kochhar, 2006; Wiklund et al., 2008). PLS‐DA has been used in distinguishing different kinds of Chinese liquors (Zhang, Yuan, Zeng, et al., 2017) and pit muds (Zhang, Yuan, Liao, & Zhang, 2017). It may therefore be of use in distinguishing different FTL flavor styles and in identifying microbes, which contribute significantly to desirable tobacco characteristics.

The network interface, in the form of a set of nodes and edges, carries meaningful statistical and structural features that shed light on the underlying rules guiding the community components and functions of the system being described (Newman, 2006). Recently, network analysis has been widely applied to reveal ecological linkages among microorganisms in complex ecosystems, such as marine water (Steele et al., 2011), soil (Barberán, Bates, Casamayor, & Fierer, 2012), and pit mud (Hu, Du, Ren, & Xu, 2016). To our knowledge, the existence of direct or indirect interactions among microbial taxa coexisting in FTLs has not been reported. Identifying and describing such interactions could clarify the ecological rules guiding community assembly within the FTL ecosystem.

## 2 MATERIALS AND METHODS

### 2.1 FTLs sampling

Samples of FTL were collected from a tobacco warehouse located in Shifang city, Sichuan province of China. The FTLs labeled as fresh flavor style and strong flavor style by sensory assessors were marked accordingly (F1, F2, F3, S1, S2, and S3). FTLs from three well‐known planting origins located in China were randomly selected for each style, and triplicate subsamples were collected and placed into each tobacco leaf storage box. Tobacco leaves approximately 20 cm from the top of the tobacco leaf storage box was removed and discarded. In total, 2 kg of leaf samples were taken from the four corners and the center of the storage box using the five‐point method. All samples were well‐mixed, transferred into sterile bags, and stored at −20°C.

### 2.2 DNA extraction and illumina MiSeq sequencing

Tobacco (25 g) was suspended in 500 ml of sterile saline and shaken for 2 hr at 200 rpm, after which the supernatant was centrifuged at 10,000 *g* for 20 min. Genomic DNA was extracted from the resulting pellet using an EZNA® Soil DNA Kit (Omega). The genomic DNA was sent to GENEWIZ Inc. for PCR amplification and sequencing of the V3‐V4 hypervariable region of 16S rRNA genes (primers: 5′‐CCT ACG GRR BGC ASC AGK VRV GAA/T3′ and 5′‐GGA CTA CNV GGG TWT CTA ATC C‐3′) and the V7‐V8 hypervariable region of 18S rRNA genes (forward primers containing the sequence: 5′‐CGW TAA CGA ACG AG‐3′ and reverse primers containing 5′‐AIC CAT TCA ATC GG‐3′).

DNA libraries, validated by Agilent 2100 Bioanalyzer (Agilent) and quantified by Qubit 2.0 Fluorometer (Invitrogen), were multiplexed and loaded on an Illumina MiSeq sequencing system according to manufacturer's instructions (Illumina). Sequencing was performed using a 2 × 250 paired‐end (PE) configuration. Image analysis and base calling were performed using the MiSeq Control Software (MCS) of the MiSeq instrument.

### 2.3 Sequence processing and data analysis

The sequencing data were processed using the QIIME platform version 1.9.1 (http://qiime.org/). The forward and reverse reads were merged according to the unique sample barcode sequence, followed by quality control processing (for 16S rRNA gene lengths between 430 and 470 bp, average length 455 bp, and for 18S rRNA gene lengths between 340 and 380 bp, average length 355 bp), and then truncated by removing the barcode and primer sequences. Qualified sequences were classified into operational taxonomic units (OTUs) at a 97% sequence identity using the clustering program VSEARCH version 1.9.6 and the Silva 132 database (https://www.arb-silva.de/) (Rognes, Flouri, Nichols, Quince, & Mahé, 2016). The phylogenetic affiliation of each sequence was analyzed by Ribosomal Database Program (RDP) Classifier at the 80% confidence level. Significant differences between the fresh flavor style and strong flavor style FTL groups were determined using SPSS software, version 19 (IBM), by one‐way analysis of variance and Duncan's multiple comparison test (*p < .*05). Alpha diversity, including Chao1 and Shannon values, was analyzed using QIIME version 1.9.1 (Caporaso et al., 2010). To reduce potential confounding effects due to uneven sampling, we randomly rarefied the OTU table to an even depth for alpha diversity analysis.

PLS‐DA and hierarchical cluster analysis (HCA) were conducted using SIMCA‐P version 13.0 (UMETRICS, Sweden) to discriminate different flavor styles of FTLs and reveal specific markers according to microbial compositions. The cross‐validated coefficient of determination, Q2, which indicates the variance captured in cross‐validation, was used as an indicator of overfitting. R2 was used to indicate the variance captured with the model (Pantsar‐Kallio, Reinikainen, & Oksanen, 2001). CoeffCS are coefficients used for interpreting how strongly Y is correlated with the systematic part of each of X‐variable. PLS‐DA of various FTLs is represented as a two‐dimensional representation of the scores (t[1] and t[2]) on the first and second PLS‐DA components.

Pairwise Spearman's rank correlations among genera with relative abundances higher than 0.1% were performed using SPSS Statistics version 19 (IBM, America). Spearman's correlation coefficients with statistical significance (*p* < .01) were considered valid co‐occurrence (or negative) events for a robust correlation (Barberán et al., 2012; Hu et al., 2016; Zhao et al., 2014). Networks were explored and visualized using the interactive platform Gephi (Bastian, Heymann, & Jacomy, 2009) based on the correlation matrix constructed by Spearman's correlations, with each node and edge representing one genus and a strong and significant correlation, respectively.

### 2.4 Nucleotide sequence accession number

The MiSeq sequences determined in this study have been deposited in the GenBank under the following accession number: PRJNA498896 and release date: 2019–11–19.

## 3 RESULTS

### 3.1 Prokaryotic Community Diversity and Structure

**Figure A1:**
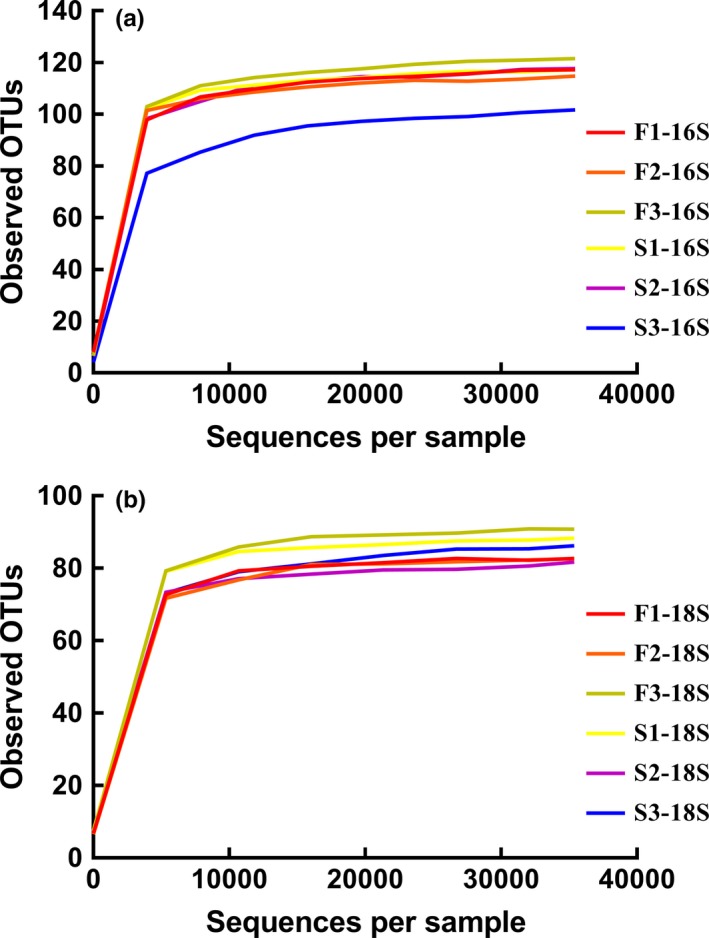
Rarefaction curve of the observed number of OTUs from samples of 16S rRNA gene (a) and 18S rRNA gene (b) at a genetic distance of 3%

In total, 344,929 qualified reads were obtained from all FTLs. Each sample contained 102 to 122 OTUs, based on 97% similarity of 16S rRNA sequences (Table 1). The rarefaction curves all reached the saturation plateau (Figure A1 a) with coverage of more than 99%, indicating that Illumina MiSeq sequencing was deep enough to represent all bacterial communities detected. The Chao1 and Shannon values in the two different groups suggested similar bacterial species richness and diversity in all samples.

**Table 1:** Diversity indices of prokaryotic and eukaryotic communities in FTLs

Sample	Prokaryotic diversity					Eukaryotic diversity				
	Qualified Sequences	OTUs	Chao1	Shannon	Coverage	Qualified Sequences	OTUs	Chao1	Shannon	Coverage
F1	69017	118	119.00	5.23	0.99	60332	83	83.00	3.94	0.99
F2	58868	115	120.00	4.87	0.99	61357	83	83.00	3.82	0.99
F3	75999	122	124.00	4.30	0.99	61380	92	92.50	4.22	0.99
S1	49342	118	121.33	5.16	0.99	55261	89	90.00	4.43	0.99
S2	42724	118	123.00	4.47	0.99	59396	83	88.00	4.46	0.99
S3	48979	102	104.14	2.50	0.99	69611	87	87.17	4.14	0.99

Note

F1 to F3 denote fresh flavor style FTLs; S1 to S3 denote strong flavor style FTLs.

**Figure 1:**
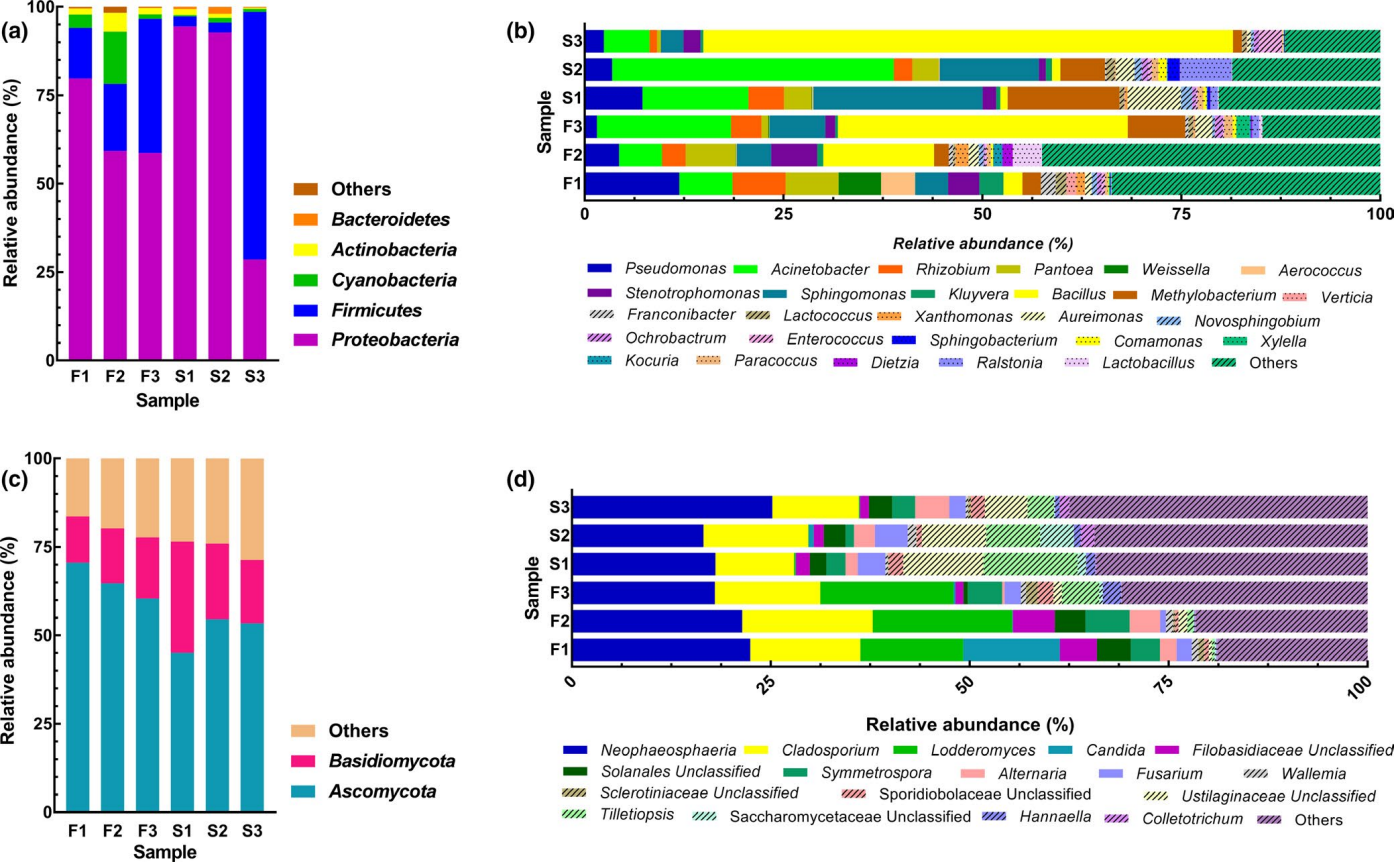
Plot of phylum and genus level relative abundances of prokaryotic and eukaryotic communities in FTLs. (a) and (b) represent prokaryotic communities at the level of phylum and genus; (c) and (d) represent eukaryotic communities at the level of phylum and genus. F1 to F3 denote fresh flavor style FTLs; S1 to S3 denote strong flavor style FTLs

The phylogenetic structure analysis indicated that the identified sequences were affiliated with five bacterial phyla: *Proteobacteria*, *Firmicutes*, *Cyanobacteria*, *Actinobacteria*, and *Bacteroidetes* (Figure 1a)*.* Based on the average relative abundance, the dominant bacterial phyla were *Proteobacteria* (68.93 ± 25.16%) and *Firmicutes* (24.49 ± 25.81%). A total of 54 bacterial genera were detected, of which 27 had a relative abundance higher than 1.0% in at least one sample (Figure 1b). The dominant bacterial genera, with relative abundance higher than 5.0% in at least one sample, were *Pseudomonas, Acinetobacter*, *Rhizobium*, *Pantoea*, *Weissella*, *Bacillus*, *Methylobacterium*, *Sphingomonas*, *Aureimonas*, and *Ralstonia*, which represented between 45.51% and 82.66% of the total abundance of each FTL sample. Compared with the proportions of the dominant genera in the strong flavor style FTLs (S1, S2, and S3), the proportions of most groups decreased slightly (*p > .*05) in the fresh flavor style FTLs (F1, F2, and F3).

### 3.2 Eukaryotic community diversity and structure

In total, 367,337 qualified reads were obtained from all FTLs (Table 1). Each sample contained 83 to 92 OTUs, based on 97% similarity of 18S rRNA sequences. Based on the rarefaction curves (Figure A1 b) and coverage values, Illumina MiSeq sequencing was found to represent all fungal communities. The diversity and richness of the fungal communities were generally lower than those of the bacterial communities.

Figure 1c shows the two main eukaryotic phyla, *Ascomycota* and *Basidiomycota*, which had average relative abundances of 58.16 ± 9.06% and 19.43 ± 6.53%, respectively. *Ascomycota* had higher relative abundances (*p < .05*) in fresh flavor style FTLs (F1, F2, and F3) than in strong flavor style FTLs (S1, S2, and S3). There were 53 eukaryotic genera across all samples, with 17 genera having a relative abundance of higher than 1.0% in at least one sample (Figure 1d). *Lodderomyces* and *Symmetrospora* had higher relative abundances (*p < .*05) in the fresh flavor style FTLs than in the strong flavor style FTLs, while unclassified Ustilaginaceae displayed the opposite trend. The dominant genera were *Neophaeosphaeria*, *Cladosporium*, *Lodderomyces*, *Tilletiopsis*, unclassified Ustilaginaceae, and *Symmetrospora* represented between 67.61% and 84.66% of the total abundance in each FTL sample.

### 3.3 PLS‐DA and HCA

**Figure 2:**
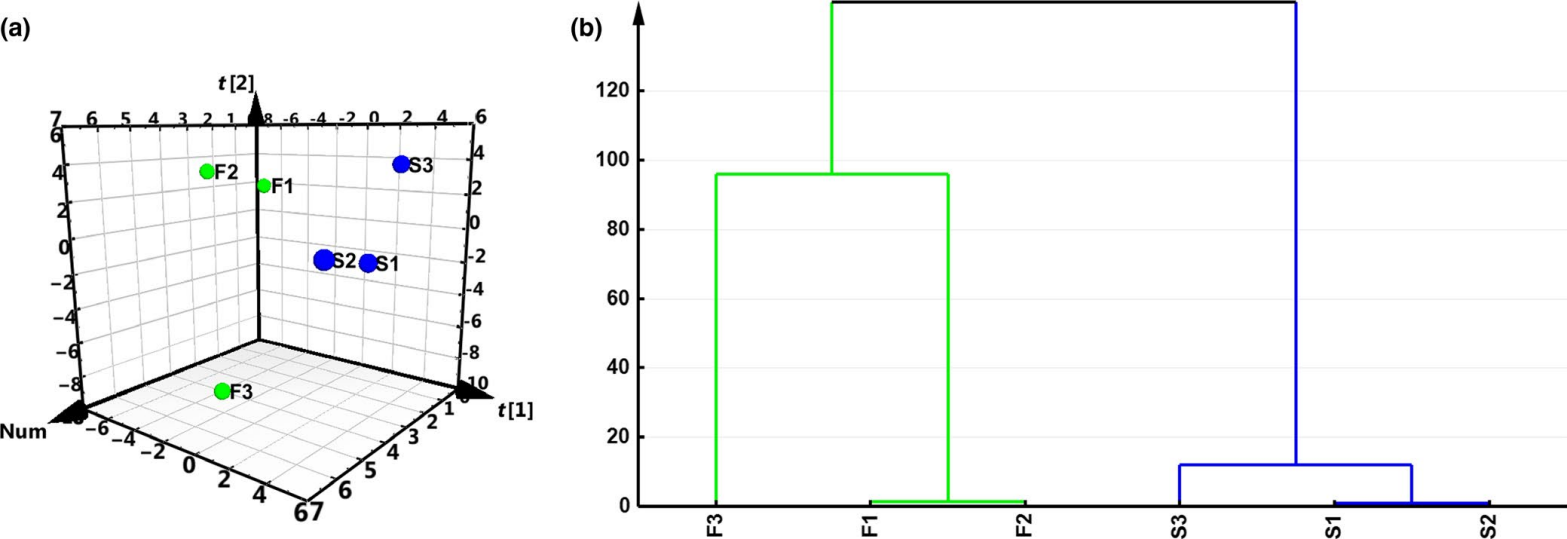
Score scatter plot of PLS‐DA (a) and dendrogram of HCA (b). PLS‐DA of various FTLs is represented as a two‐dimensional representation of the scores (t[1] and t[2]) on the first and second PLS‐DA components. F1 to F3 denote the fresh flavor style FTLs; S1 to S3 denote strong flavor style FTLs

PLS‐DA was used to construct a statistical model for FTL discrimination and classification, and two significant principal components of the total variance in data matrix were extracted. The R2 and Q2 were 0.997 and 0.775, respectively, which meant that a total of 99.7% dummy Y variable per class, and 77.5% overall cross‐validated *R* ^2^ for these two components. The data indicated that the PLS‐DA model was suitable for this research. Fresh flavor style and strong flavor style FTL groups were clearly separated on the score scatter plot, with the fresh flavor style group located on the left side of the plot and the strong flavor style group located on the right side (Figure 2a). The coefficients refer to the PLS‐DA model being rewritten as a regression model. CoeffCS (coefficient values between variables and samples of significant first and second principal components) are shown in Table 2. *Xanthomonas*, *Franconibacter*, *Massilia*, *Quadrisphaera*, *Staphylococcus*, *Cladosporium*, *Lodderomyces*, *Symmetrospora*, *Golovinomyces*, and *Dioszegia* were significantly positively correlated with DA(1) (fresh flavor style FTL group). *Xenophilus*, *Fusarium*, unclassified Ustilaginaceae, *Tilletiopsis*, *Cryphonectria*, *Colletotrichum*, and *Cyanodermella* were significantly positively correlated with DA(2) (strong flavor style FTL group). HCA also identified two groups of samples (Figure 2b), the first consisting of samples F1, F2, and F3, and the second of samples S1, S2, and S3, which was consistent with the PLS‐DA.

**Table 2:** Coefficient values between variables and group†

Variable	CoeffCS[1]		CoeffCS[2]	
	DA(1)	DA(2)	DA(1)	DA(2)
*Stenotrophomonas*	0.020164*	−0.020164‡	0.014278	−0.014280
*Xanthomonas*	0.022872‡	−0.022872‡	0.018785‡	−0.018785‡
*Franconibacter*	0.022677‡	−0.022677‡	0.023343‡	−0.023343‡
*Brachybacterium*	0.020946‡	−0.020946‡	0.017670	−0.017670‡
*Massilia*	0.028344‡	−0.028344‡	0.032326‡	−0.032326‡
*Aeromicrobium*	0.020618	−0.020620	0.024559‡	−0.024559‡
*Roseomonas*	−0.019860‡	0.019860‡	−0.018810	0.018809
*Quadrisphaera*	0.022342‡	−0.022342‡	0.020474‡	−0.020474‡
*Xenophilus*	−0.028989‡	0.028989‡	−0.039998‡	0.039998‡
*Staphylococcus*	0.027194‡	−0.027194‡	0.034612‡	−0.034612‡
*Sporolactobacillus*	0.023550	−0.023550	0.033789‡	−0.033789‡
*Cladosporium*	0.025311‡	−0.025311‡	0.026734‡	−0.026734‡
*Lodderomyces*	0.032772‡	−0.032772‡	0.039731‡	−0.039731‡
unclassified Filobasidiaceae	0.021517‡	−0.021517‡	0.016375	−0.016380
*Symmetrospora*	0.027974‡	−0.027974‡	0.030922‡	−0.030922‡
*Fusarium*	−0.024946‡	0.024946‡	−0.024955‡	0.024955‡
*Vishniacozyma*	0.021165‡	−0.021165‡	0.019052	−0.019050
*Aureobasidium*	0.015633‡	−0.015633‡	0.007527	−0.007530
*Golovinomyces*	0.027221‡	−0.027221‡	0.026030‡	−0.026030‡
unclassified Ustilaginaceae	−0.030978‡	0.030978‡	−0.036197‡	0.036197‡
*Dioszegia*	0.025596‡	−0.025596‡	0.028663‡	−0.028663‡
*Tilletiopsis*	−0.022887‡	0.022887‡	−0.022104‡	0.022104‡
*Taphrina*	0.025651	−0.025650‡	0.036222‡	−0.036220‡
*Cryphonectria*	−0.024840‡	0.024837‡	−0.02856‡	0.028563‡
*Rhizoctonia*	−0.025910‡	0.025914‡	−0.027720	0.027717
*Colletotrichum*	−0.023750‡	0.023760‡	−0.028350‡	0.028349‡
*Cyanodermella*	−0.029790‡	0.029789‡	−0.034680‡	0.034678‡

^†^ CoeffCS are coefficients used for interpreting the influence of the variables X on Y. CoeffCS[1] and CoeffCS[2] represent significant principal component 1 and 2, respectively. DA(1) denotes the fresh flavor style FTL group (F1 to F3); DA(2) denotes strong flavor style FTL group (S1 to S3).

^‡, *^ Coefficient is significant (above the noise).

### 3.4 Correlation network description

**Figure 3:**
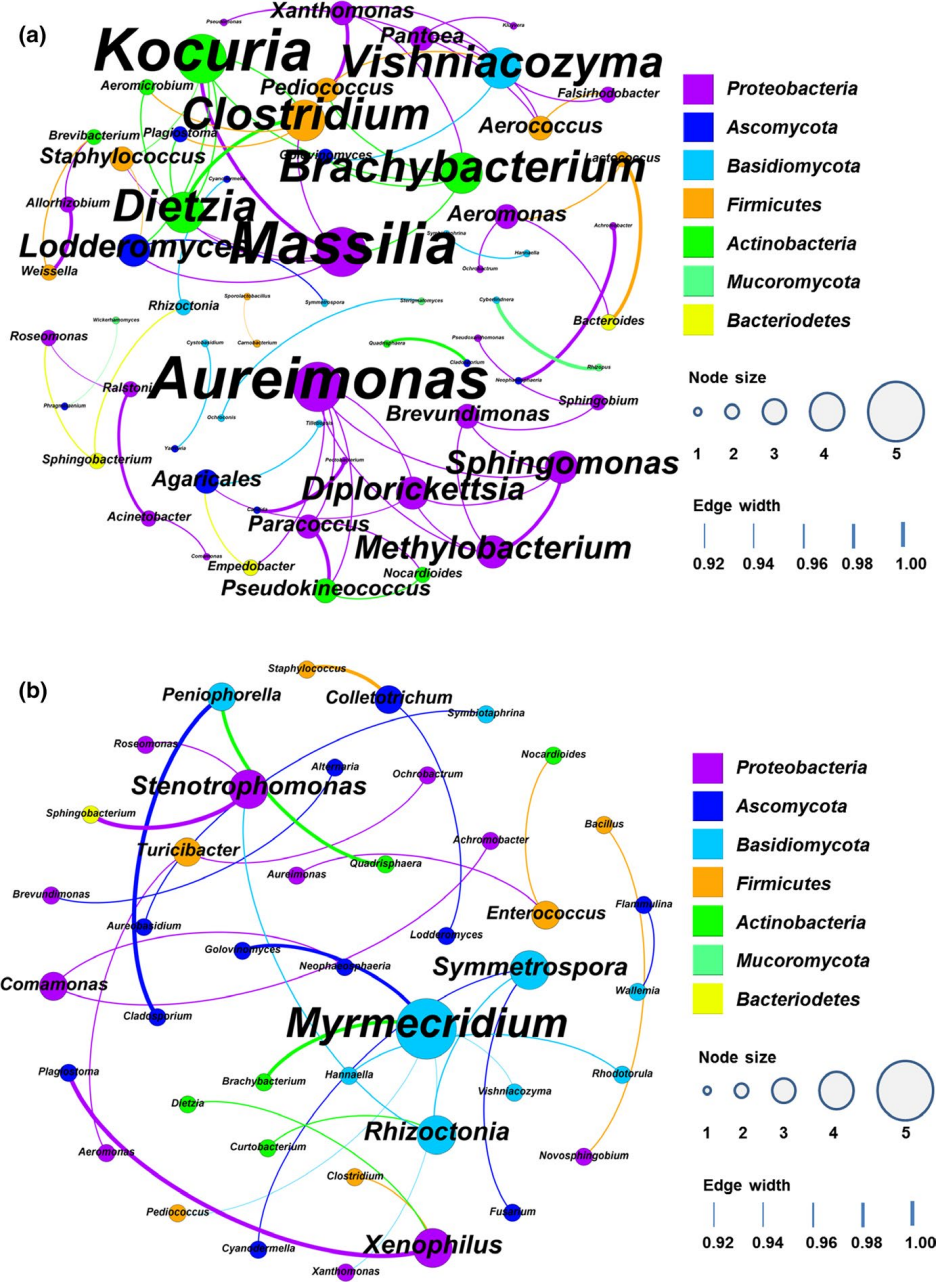
Network of co‐occurring microbial genera in FTLs based on correlation analysis, positive and negative correlation. (a) positive correlation network; (b) negative correlation network. For each panel, the size of each node is proportional to the number of connections, nodes of the same color are affiliated with the same phylum, and the thickness of each connection between two nodes is proportional to the value of Spearman's correlation coefficients with statistical significance (*p* < .01)

The co‐occurrence patterns of FTL microorganisms were explored based on strong and significant correlations (*p* < .01), and a total of 64 nodes (genera) and 72 edges (pairs of significant and robust correlations) were found (Figure 3a). The modularity index was 0.797 (*>*0.4), suggesting that the network had a modular structure. At the phylum level, *Proteobacteria*, *Ascomycota*, *Basidiomycota*, *Firmicutes*, and *Actinobacteria* accounted for 35.94%, 15.62%, 14.06%, 12.5%, and 12.5% of all nodes, respectively. The average degree (edges per node) was 2.25. There were seven hubs (highly connected genera, degree ≥ 5), including *Aureimonas*, *Kocuria*, *Massilia*, *Brachybacterium, Clostridium, Dietzia*, and *Vishniacozyma*. Genera from different phyla (interphylum) had a high co‐occurrence incidence (69.4%, ratio of targeted edges to total edges). Between all pairs of any two phyla, the incidence of co‐occurrence between *Proteobacteria* and *Firmicutes*, and between *Proteobacteria* and *Actinobacteria,* was the highest, at up to 11.1%. *Massilia* was significantly and positively correlated with *Kocuria,* and *Clostridium* with *Dietzia*. The incidence of co‐occurrence within a phylum (intraphylum) was 30.6% and was observed among genera from the phyla *Proteobacteria* (22.2%) and *Actinobacteria* (5.6%). *Methylobacterium* was significantly and positively correlated with *Sphingomonas.*

Moreover, a total of 30 pairs of significant and robust negative correlations were identified from 43 genera (Figure 3b). The modularity index was 0.88 (*>*0.4), suggesting that the network had a modular structure. At the phylum level, *Proteobacteria*, *Ascomycota,* and *Basidiomycota* accounted for 25.58%, 25.58%, and 20.93% of all nodes, respectively. The average degree (edges per node) was 1.395, and the highest degree was 5 (hubs, *Myrmecridium*). Genera from different phyla (interphylum) had a high incidence of negative occurrence (80.0%, ratio of targeted edges to total edges).

## 4 DISCUSSION

In this study, Illumina MiSeq sequencing based on 16S rRNA and 18S rRNA genes was used to investigate the diversity and structure of the prokaryotic and eukaryotic communities associated with FTLs. Based on their microbial community compositions, the differences between fresh and strong flavor style FTLs were explored, as along with the co‐occurrence patterns of FTL microorganisms in the individual communities. *Firmicutes*, *Proteobacteria*, *Actinobacteria*, and *Bacteroidetes* were abundant in FTLs. However, the most dominant phylum was either *Proteobacteria* or *Firmicutes*, corroborating the results of previous studies (Huang et al., 2010; Su et al., 2011; Wang et al., 2018). Specifically, Huang et al. (2010) and Su et al. (2011) indicated that *Proteobacteria* was the dominant phylum of Zimbabwe FTLs and K326 FTLs, while Wang et al. (2018) found that *Firmicutes* was the dominant phylum of FTLs.

Compared with PCR‐DGGE, 16S rRNA gene clone libraries sequencing, and pyrosequencing, Illumina MiSeq sequencing with a higher sequence output could unveil more information about microbial community (Hirai, Nagai, & Hidaka, 2017). In our study, the Illumina MiSeq sequencing results showed that 61.72% of the prokaryotes belonged to the genera *Pseudomonas, Bacillus*, *Methylobacterium*, *Acinetobacter*, and *Sphingomonas*. These genera are reported to be important contributors to nicotine degradation, or to the formation of representative flavor compounds. They are also used as biocontrol agents. Although bacterial genera varied from different resources and sequencing methods (Liu, He, et al., 2015; Liu, Ma, et al., 2015), *Pseudomonas* and *Bacillus* are the dominant genera in most of the FTL samples (Huang et al., 2010; Su et al., 2011; Wang et al., 2018; Zhao et al., 2007), as shown in our study, and functionally important contributors in the process of FTL aging (Huang et al., 2010; Wang et al., 2018). *Pseudomonas* strains isolated from tobacco leaves and tobacco soils have been reported to be nicotine‐degrading. Isolates include *Pseudomonas* sp. HF‐1 (Ruan, Min, Peng, & Huang, 2005), *P. putida* S16 (Wang, Liu, Tang, Meng, & Xu, 2007), *Pseudomonas* sp. Nic22 (Chen et al., 2008), *Pseudomonas* sp. ZUTSKD (Zhong et al., 2010), and *P. stutzeri* ZCJ (Zhao et al., 2012). Nicotine degradation pathways in *Pseudomonas* species can be classified into two categories, depending on whether metabolites are directed into the tricarboxylic acid cycle (TAC). Nicotine can be converted into N‐methylmyosmine, cotinine, or nornicotine, which is then converted into maleamic acid, and finally fumaric acid in the TAC. Alternatively, nicotine may be converted into nicotyrine, which is not directed into the TAC (Li, Duan, Zhang, & Yang, 2010; Ruan et al., 2005; Tang et al., 2008, 2009, 2011; Wang, Yang, Min, & Lv, 2009).

*Bacillus* species fulfill different functions in FTLs. Some species are considered endophytic and/or beneficial to plants, including tobacco, and have been reported to be the functional microorganism in the promotion of tobacco fermentation and formation of aged flavor compounds (English et al., 1967; Huang et al., 2010). English et al. (1967) revealed that *B. subtilis* and *B. circulans* could hasten the development of desirable flavors and improve the smoking qualities of cigar tobacco. *B. thuringiensis,* which produces bipyramidal crystals, is present on the tobacco leaf surface and can control insect pests that affect stored tobacco (Kaelin & Gadani, 2000). *B. subtilis* has a strong ability to control the effects of tobacco black shank (Han et al., 2016) and displayed an antagonistic effect against *Verticillium dahliae*, which causes verticillium wilt (Berg & Ballin, 1994).

Similar to *B. subtilis*, *Sphingomonas* has an antagonistic effect on *Verticillium dahliae* (Berg & Ballin, 1994), but has the additional ability to degrade a wide variety of dimeric lignin compounds into a series of flavor compounds (Masai, Katayama, Nishikawa, & Fukuda, 1999). *Sphingomonas* abundance was 4% in the 16S rRNA clone library of Zimbabwe tobacco (Su et al., 2011). *Acinetobacter* and *Sphingomonas* isolated from soil tobacco waste are able to degrade nicotine (Wang et al., 2011). *Methylobacterium* strains, which are frequently encountered as endophytes, degrade one‐carbon compounds such as methanol and methylamine, and are capable of forming biofilms, producing quorum‐sensing signals, and resisting heavy metal and other stresses (Ardanov, Sessitsch, Häggman, Kozyrovska, & Pirttila, 2012; Rossetto et al., 2011).

*Neophaeosphaeria* and *Cladosporium*, from the *Ascomycota*, were dominant fungal genera in FTLs, although *Lodderomyces*, *Candida*, unclassified Ustilaginaceae, and *Tilletiopsis* were also present at high relative abundances (>10%) in some samples. However, the specific functions of these genera during tobacco leaf fermentation and flavor formation are not well‐understood. *Cladosporium* can produce γ‐decalactone (Berger, 2015). It can also contribute to lignin and cellulose‐degradation, as it produces laccase and cellulase (Jin et al., 2012), and may consequently play a flavor‐enhancing role during FTL fermentation. *Tilletiopsis* produce hydrolytic enzymes and antifungal compounds, which are effective against powdery mildew fungi (Urquhart & Punja, 2002); this genus may therefore serve as a biocontrol agent.

The relative abundance of microorganisms varied between different FTL samples, and even within the same flavor style FTLs, relative abundance changed. The planting area (including edaphic factors, climatic factors, and biologic factors) may directly cause differences in the microbial communities among the same flavor style FTLs. By one‐way analysis of variance and Duncan's multiple comparison test, there was only one phylum (*Ascomycota*) and three (*Lodderomyces*, *Symmetrospora*, and unclassified Ustilaginaceae) out of 107 genera that were significantly different (*p < .*05) between fresh flavor style FTLs and strong flavor style FTLs. However, the boundary separating fresh and strong flavor style FTLs was not obvious and did not allow for straightforward discrimination or classification. Therefore, in order to distinguish between fresh and strong flavor style FTLs, the microbial data had to be analyzed using metrology tools. Analysis of the microbial relative abundances by PLS‐DA and HCA indicated that samples could be separated into fresh and strong flavor groups and that some genera could be used as markers for the discrimination of samples. The results of our study agree with those of the sensory evaluation methods traditionally used to classify FTLs.

Further investigation was conducted by correlation‐based network analysis. The less‐related interphylum genera had much higher co‐occurrence ratios than intraphylum genera in FTLs, a finding that has also been observed in activated sludge (Ju, Xia, Guo, Wang, & Zhang, 2014) and pit muds (Hu et al., 2016). This phenomenon can be attributed to two factors: phylogenetic overdispersion of all biological communities, and the effect of negative interactions on the community assembly (Horner‐Devine & Bohannan, 2006; Ju et al., 2014; Slingsby & Verboom, 2006). The co‐occurrence patterns revealed that community members may share niche spaces and have synergetic relationships in FTLs (Barberán et al., 2012). Based on co‐occurrence analysis there existed eight main hubs that may play important roles in ecosystem stability: *Aureimonas*, *Kocuria*, *Massilia*, *Brachybacterium, Clostridium, Dietzia*, *Vishniacozyma*, and *Myrmecridium* (Peura, Bertilsson, Jones, & Eiler, 2015). Genera from the main hubs were endophytes (*Aureimonas*, Ikeda et al., 2010; *Myrmecridium*, Tan et al., 2012) and plant‐growth‐promoting rhizobacteria (*Brachybacterium*, Gontia, Kavita, Schmid, Hartmann, & Jha, 2011, Jiang et al., 2018; *Dietzia*, Bharti, Pandey, Barnawal, Patel, & Kalr, 2016). It is possible that *Massilia* (Myeong, Seong, Kim, & Sul, 2016; Ofek, Hadar, & Minz, 2012; Xu et al., 2016), *Vishniacozyma* (Gramiscia, Lutzb, Lopesa, & Sangorrína, 2018), and *Clostridium* (Doi et al., 1998; Murashima, Kosugi, & Doi, 2003) produce antibiotic compounds or enzymes to maintain their own niches in FTLs, but this requires further confirmation.

Although the number of samples (*n* = 3) was statistically significant and results of previous studies (Huang et al., 2010; Su et al., 2011; Wang et al., 2018) were corroborated, the sample size is still small in this study. More samples of fresh and strong flavor style FTLs from different batches and sources should be considered to verify the reliability and reproducibility. Illumina MiSeq sequencing based on 16S rRNA and 18S rRNA genes provided the diversity and structure of the prokaryotic and eukaryotic communities associated with FTLs; however, amplicon‐based studies could not identify viable organisms. Thus, omics technologies, including genomics, transcriptomics, proteomics, metabolomics, and fluxomics, should be utilized to intensively study genetic, protein, and product information related to the microbial metabolism, and the mechanisms producing patterns of community coexistence, as the complex structure and community of microbes in FTLs is complex, and several correlations of the microbial taxa. Moreover, some specific species, including endophytes, plant‐growth‐promoting rhizobacteria, and other abundance microbes isolated from FTLs, should be studied for their function in FTLs, as they might play essential roles in stabilizing inhabiting microbial community and producing beneficial substances for FTLs.

## 5 CONCLUSION

We used Illumina MiSeq sequencing to analyze the microbiota associated with fresh and strong flavor style FTLs. Bacterial and fungal community compositions and diversities were analyzed, distinctions among FTLs of different types were revealed, and correlations between microbial taxa within FTL ecosystems were described. The dominant microbes came from seven genera, including *Pseudomonas, Bacillus*, *Methylobacterium*, *Acinetobacter*, *Sphingomonas*, *Neophaeosphaeria*, and *Cladosporium*, belonging to bacterial phyla *Firmicutes* and *Proteobacteria*, and the fungal phyla *Ascomycota*. *Xanthomonas*, *Franconibacter*, *Massilia*, *Quadrisphaera*, *Staphylococcus*, *Cladosporium*, *Lodderomyces*, *Symmetrospora*, *Golovinomyces*, and *Dioszegia* were significantly positively correlated with the fresh flavor style FTL group, while *Xenophilus*, *Fusarium*, unclassified Ustilaginaceae, *Tilletiopsis*, *Cryphonectria*, *Colletotrichum*, and *Cyanodermella* were significantly positively correlated with the strong flavor style FTL group. Additionally, endophytes and rhizobacteria, including *Aureimonas*, *Kocuria*, *Massilia*, *Brachybacterium, Clostridium, Dietzia*, and *Vishniacozyma*, combined multiple niches in FTLs, which might be destroyed by *Myrmecridium.* These findings represent a step forward in revealing microbial diversities, understanding differences in microbial community structure, and uncovering the stable microbial communities associated with fresh and strong flavor FTLs.

## CONFLICT OF INTEREST

None declared.

## AUTHOR CONTRIBUTIONS

Dongliang Li and Zhongyang Ding designed the concept. Qianying Zhang performed formal analysis. Dongliang Li and Zhongyang Ding contributed to funding acquisition. Qianying Zhang and Zongze Geng performed investigation. Qianying Zhang and Zongze Geng prepared the original draft. Qianying Zhang reviewed and edited the manuscript.

## ETHICS STATEMENT

None required.

## Data Availability

The datasets generated for this study can be found in GenBank under the accession number PRJNA498896: https://www.ncbi.nlm.nih.gov/bioproject/PRJNA498896.
